# Supplementary material for: Room temperature magnetization switching in topological insulator-ferromagnet heterostructures by spin-orbit torques
Source: Nat Commun. 2017 Nov 8;8:1364. doi: 10.1038/s41467-017-01583-4 (PMC5677620; doi:10.1038/s41467-017-01583-4)
Supplement: Supplementary file 1 — Supplementary information [file 41467_2017_1583_MOESM1_ESM.pdf]

Supplementary Figures:

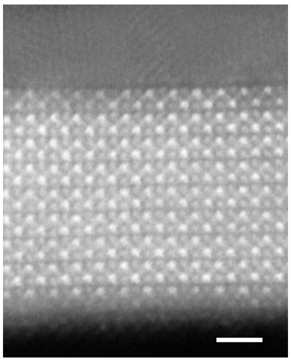

Supplementary Figure 1. HAADF-STEM image of the Bi_2Se_3/NiFe (Py) heterostructures on Al_2O_3 substrate. The uniform and good layered structure in Bi_2Se_3 and a clean and smooth interface between Bi_2Se_3 and Py layer are observed. The white scale bar is 2 nm.

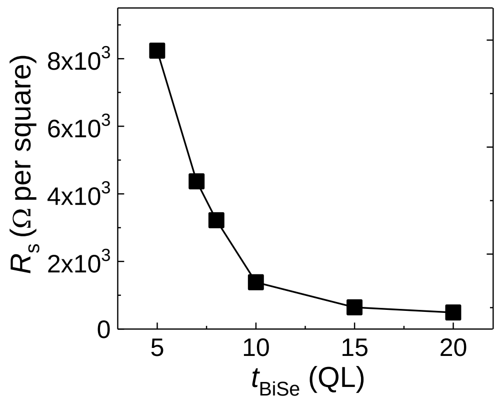

Supplementary Figure 2. Bi_2Se_3 film electrical transport property. Sheet resistance, R_s, for different Bi_2Se_3 thicknesses measured at room temperature.

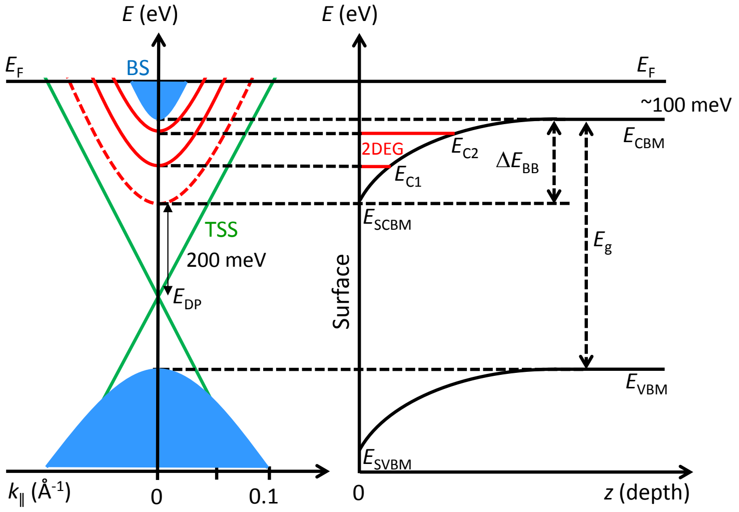

Supplementary Figure 3. Schematic of energy dispersion in *k*-space and band bending at Bi_2Se_3 surfaces. Discrete subbands are formed in 2DEG at the Bi_2Se_3 surface due to the quantum confinement effect. For clarity the Rashba splitting is not illustrated in the 2DEG subbands.

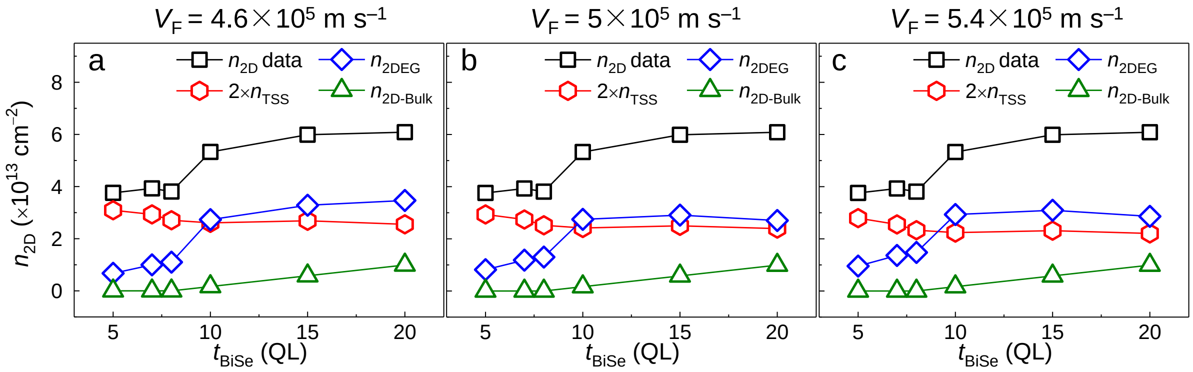

Supplementary Figure 4. Estimated n_{2D} with varying the value of V_F. a, Sheet carrier concentrations of TSS (n_{TSS}), 2DEG (n_{2DEG}) and bulk ($n_{2D\text{-}Bulk}$) estimated with $V_F = 4.6\times10^5$ m s^{-1}. **b**, with $V_F = 5\times10^5$ m s^{-1}. **c**, with $V_F = 5.4\times10^5$ m s^{-1}, by fixing $m^* = 0.14\ m_0$, $E_{SCBM} - E_{DP} = 200$ meV and $E_F - E_{CBM} = 100$ meV for different Bi_2Se_3 thicknesses at room temperature. The data with black squares represent the measured sheet carrier concentrations in Bi_2Se_3 films at room temperature.

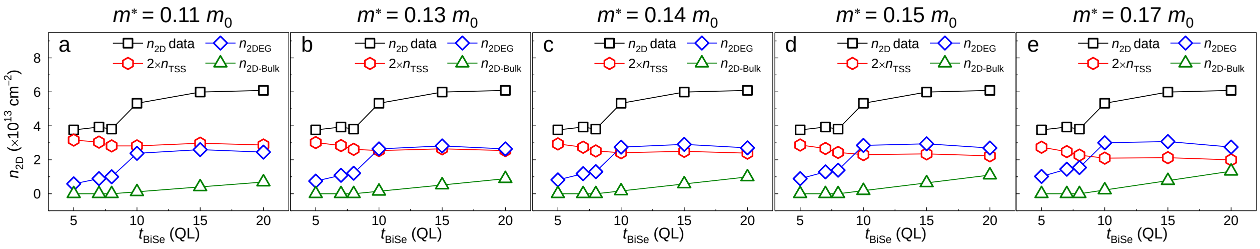

Supplementary Figure 5. Estimated n_{2D} with varying the value of m^*. a, Sheet carrier concentrations of TSS (n_{TSS}), 2DEG (n_{2DEG}) and bulk ($n_{2D\text{-}Bulk}$) estimated with $m^* = 0.11m_0$. **b**, with $m^* = 0.13m_0$. **c**, with $m^* = 0.14m_0$. **d**, with $m^* = 0.15m_0$. **e**, with $m^* = 0.17m_0$, by fixing $V_F = 5\times10^5$ m s^{-1}, $E_{SCBM} - E_{DP}$ = 200 meV and $E_F - E_{CBM}$ = 100 meV for different Bi_2Se_3 thicknesses at room temperature. The data with black squares represent the measured sheet carrier concentrations in Bi_2Se_3 films at room temperature.

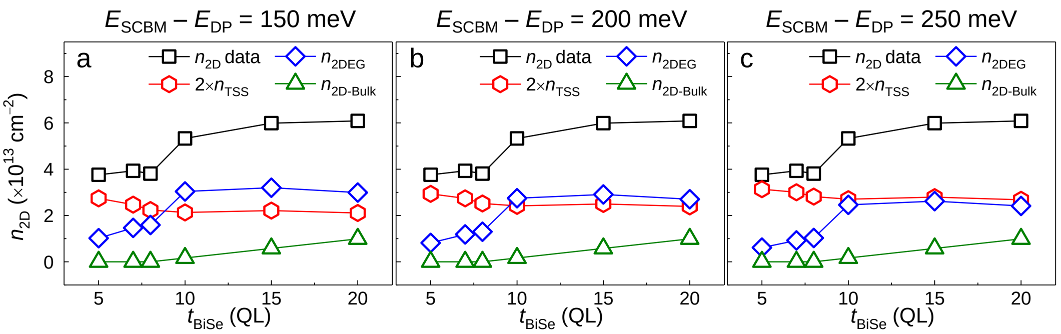

Supplementary Figure 6. Estimated n_{2D} with varying the value of ($E_{SCBM} - E_{DP}$). a, Sheet carrier concentrations of TSS (n_{TSS}), 2DEG (n_{2DEG}) and bulk ($n_{2D\text{-}Bulk}$) estimated with ($E_{SCBM} - E_{DP}$) = 150 meV. **b**, with ($E_{SCBM} - E_{DP}$) = 200 meV. **c**, with ($E_{SCBM} - E_{DP}$) = 250 meV, by fixing $V_F = 5\times10^5$ m s^{-1}, $m^* = 0.14\ m_0$ and $E_F - E_{CBM}$ = 100 meV for different Bi_2Se_3 thicknesses at room temperature. The data with black squares represent the measured sheet carrier concentrations in Bi_2Se_3 films at room temperature.

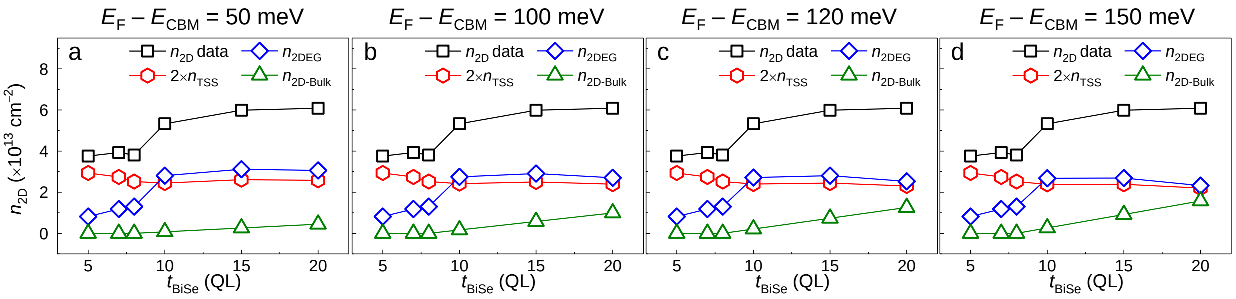

Supplementary Figure 7. Estimated n_{2D} with varying the value of (E_F – E_{CBM}). a, Sheet carrier concentrations of TSS (n_{TSS}), 2DEG (n_{2DEG}) and bulk ($n_{2D\text{-}Bulk}$) estimated with (E_F – E_{CBM}) = 50 meV. **b**, with (E_F – E_{CBM}) = 100 meV. **c**, with (E_F – E_{CBM}) = 120 meV. **d**, with (E_F – E_{CBM}) = 150 meV, by fixing $V_F = 5\times10^5$ m s^{-1}, $m^* = 0.14\ m_0$ and $E_{SCBM} - E_{DP}$ = 200 meV for different Bi_2Se_3 thicknesses at room temperature. The data with black squares represent the measured sheet carrier concentrations in Bi_2Se_3 films at room temperature.

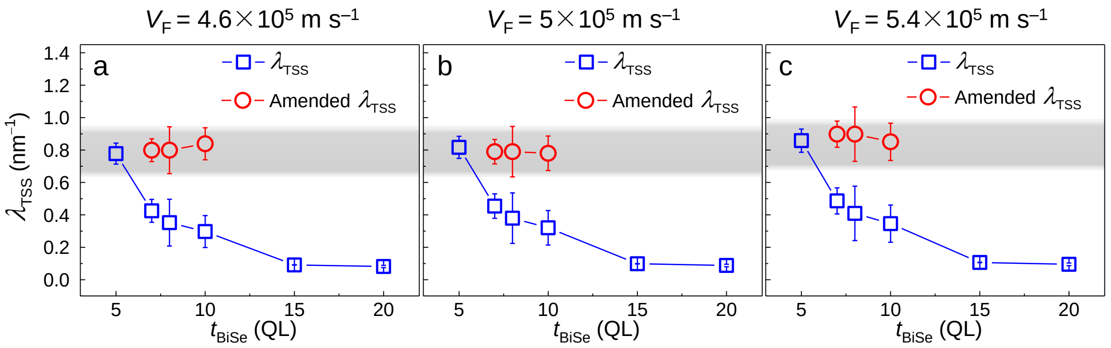

Supplementary Figure 8. Estimated interface SOT efficiency (λ_{TSS} and amended λ_{TSS}) with varying the value of V_F. a, Interface SOT efficiency estimated with $V_F = 4.6\times10^5$ m s^{-1}. **b**, with $V_F = 5\times10^5$ m s^{-1}. **c**, with $V_F = 5.4\times10^5$ m s^{-1}, by fixing $m^* = 0.14\ m_0$, $E_{SCBM} - E_{DP} =$ 200 meV and $E_F - E_{CBM} = 100$ meV for different Bi_2Se_3 thicknesses at room temperature. The amended λ_{TSS} denoted by red circles represents the interface SOT efficiency from TSS after excluding the opposite 2DEG contribution. The error bars are the standard deviation from three devices at each Bi_2Se_3 thickness.

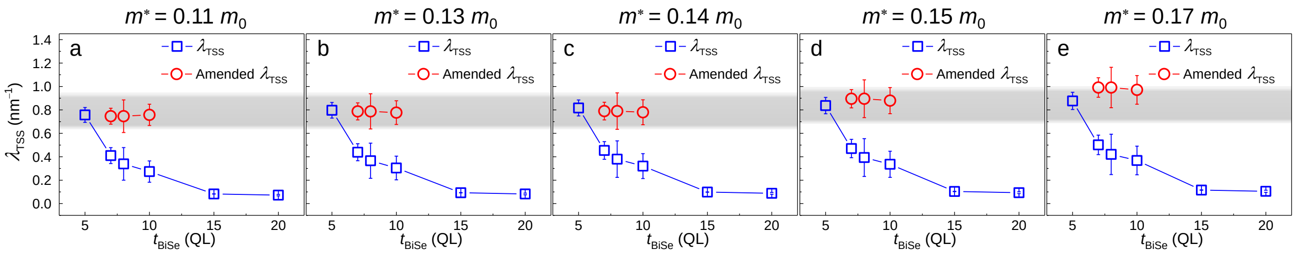

Supplementary Figure 9. Estimated interface SOT efficiency (λ_{TSS} and amended λ_{TSS}) with varying the value of m^*. a, Interface SOT efficiency estimated with m^* = 0.11m_0. **b**, with m^* = 0.13m_0. **c**, with m^* = 0.14m_0. **d**, with m^* = 0.15m_0. **e**, with m^* = 0.17m_0, by fixing V_F = 5×10^5 m s^{-1}, $E_{SCBM} - E_{DP}$ = 200 meV and $E_F - E_{CBM}$ = 100 meV for different Bi_2Se_3 thicknesses at room temperature. The amended λ_{TSS} denoted by red circles represent the interface SOT efficiency from TSS after excluding the opposite 2DEG contribution. The error bars are the standard deviation from three devices at each Bi_2Se_3 thickness.

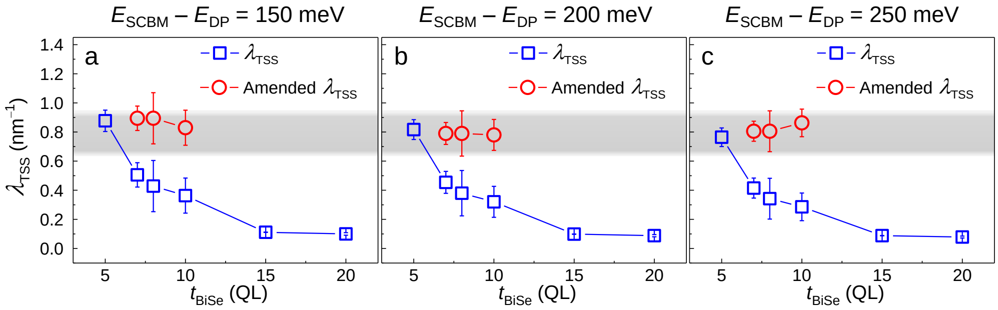

Supplementary Figure 10. Estimated interface SOT efficiency (λ_{TSS} and amended λ_{TSS}) with varying the value of $E_{SCBM} - E_{DP}$. a, Interface SOT efficiency estimated with $E_{SCBM} - E_{DP}$ = 150 meV. **b**, with $E_{SCBM} - E_{DP}$ = 200 meV. **c**, with $E_{SCBM} - E_{DP}$ = 250 meV, by fixing $V_F = 5\times10^5$ m s^{-1}, $m^* = 0.14\ m_0$ and $E_F - E_{CBM}$ = 100 meV for different Bi_2Se_3 thicknesses at room temperature. The amended λ_{TSS} denoted by red circles represent the interface SOT efficiency from TSS after excluding the opposite 2DEG contribution. The error bars are the standard deviation from three devices at each Bi_2Se_3 thickness.

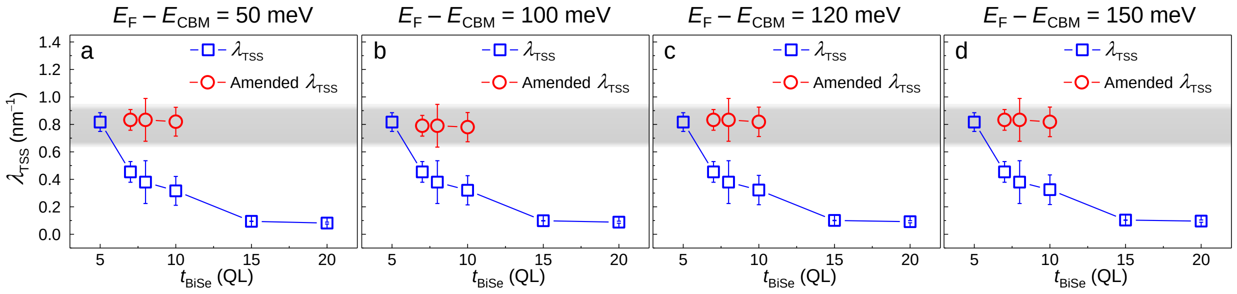

Supplementary Figure 11. Estimated interface SOT efficiency (λ_{TSS} and amended λ_{TSS}) with varying the value of $E_F - E_{CBM}$. a, Interface SOT efficiency estimated with $E_F - E_{CBM}$ = 50 meV. **b**, with $E_F - E_{CBM}$ = 100 meV. **c**, with $E_F - E_{CBM}$ = 120 meV. **d**, with $E_F - E_{CBM}$ = 150 meV, by fixing $V_F = 5\times10^5$ m s^{-1}, $m^* = 0.14\ m_0$ and $E_{SCBM} - E_{DP}$ = 200 meV for different Bi_2Se_3 thicknesses at room temperature. The amended λ_{TSS} denoted by red circles represent the interface SOT efficiency from TSS after excluding the opposite 2DEG contribution. The error bars are the standard deviation from three devices at each Bi_2Se_3 thickness.

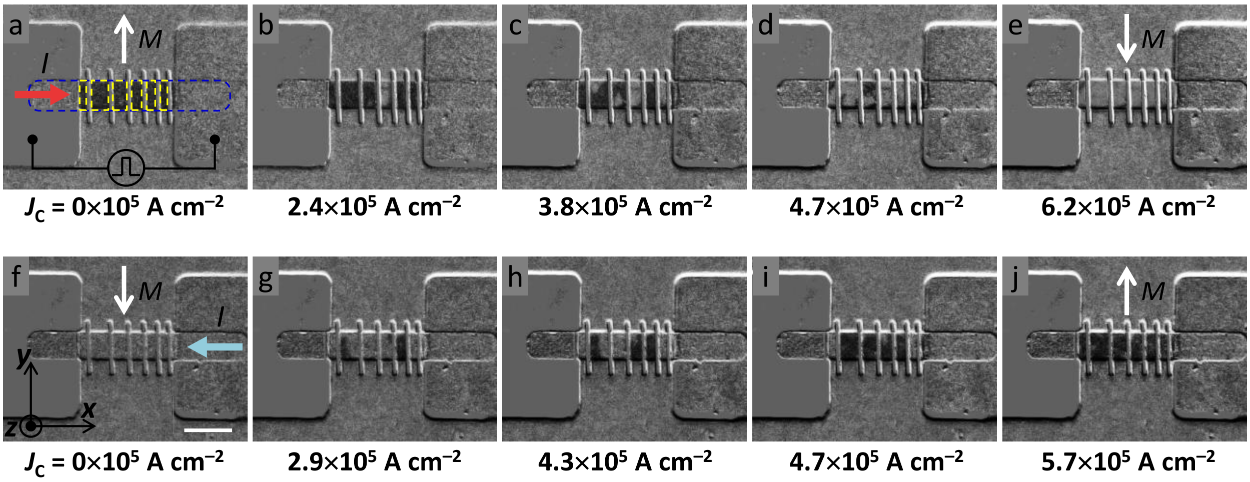

Supplementary Figure 12. MOKE images of SOT driven magnetization switching in Bi_2Se_3/Py at zero external magnetic field and room temperature. a-e, MOKE images for SOT driven magnetization switching using a pulsed current I along the +x-axis with increasing the current density J_C denoted underneath the corresponding image. **f-j**, MOKE images for SOT driven magnetization switching by I along the –x-axis. The dark (light) contrast shows the magnetization along the +y (–y)-axis, which is also indicated by the white arrows in (**a**), (**e**), (**f**) and (**j**). The white scale bar is 20 μm.

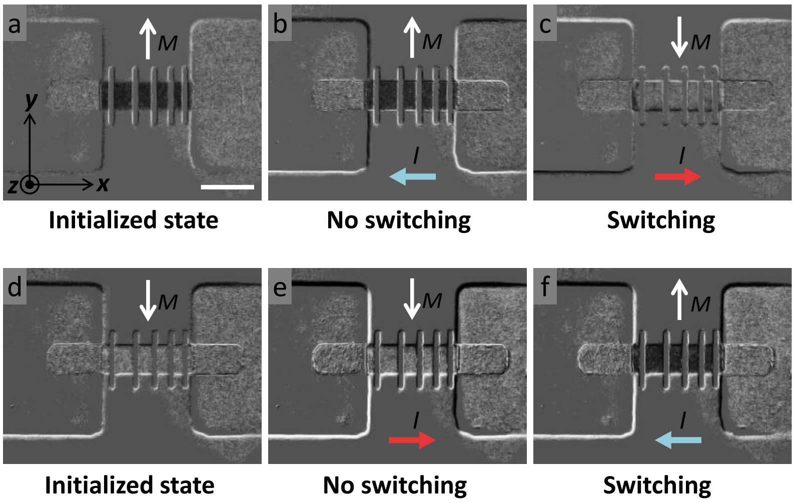

Supplementary Figure 13. Current polarity dependent magnetization switching in Bi_2Se_3/Py at zero external magnetic field and room temperature. a, Initialized state with the Py magnetization along +*y*-axis. **b-c**, Current induced magnetization switching from +*y* to –*y*-axis is only induced by a pulsed current along the +*x*-axis in the absence of an external magnetic field. **d**, Initialized state with magnetization along the –*y*-axis. **e-f**, Current induced magnetization switching from –*y* to +*y*-axis is only induced by a pulsed current along the –*x*-axis. The current channel of Bi_2Se_3/Py is 12 μm wide. The white arrows represent the Py magnetization direction in each case. The white scale bar is 20 μm.

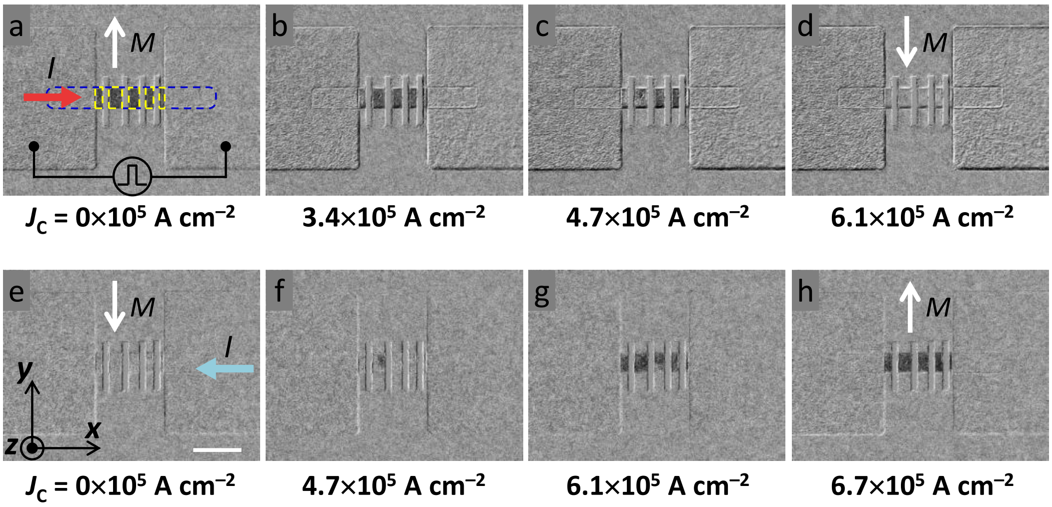

Supplementary Figure 14. MOKE images of SOT driven magnetization switching in Bi_2Se_3/Cu (1 nm)/Py at zero external magnetic field and room temperature. a-d, MOKE images for SOT driven magnetization switching using a pulsed current I along the $+x$-axis with increasing the current density J_C denoted underneath the corresponding image. **e-h**, MOKE images for SOT driven magnetization switching by I along the $-x$-axis. The dark (light) contrast shows the magnetization along the $+y$ ($-y$)-axis, which is also indicated by the white arrows in (**a**), (**d**), (**e**) and (**h**). The white scale bar is 20 μm.

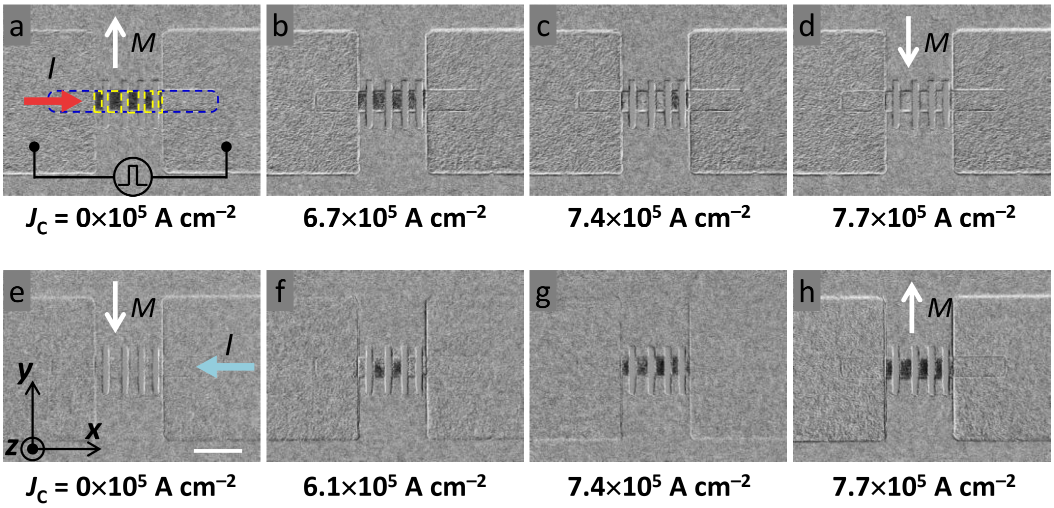

Supplementary Figure 15. MOKE images of SOT driven magnetization switching in Bi_2Se_3/Cu (2 nm)/Py at zero external magnetic field and room temperature. a-d, MOKE images for SOT driven magnetization switching using a pulsed current *I* along the +*x*-axis with increasing the current density J_C denoted underneath the corresponding image. **e-h**, MOKE images for SOT driven magnetization switching by *I* along the –*x*-axis. The dark (light) contrast shows the magnetization along the +*y* (–*y*)-axis, which is also indicated by the white arrows in (**a**), (**d**), (**e**) and (**h**). The white scale bar is 20 μm.

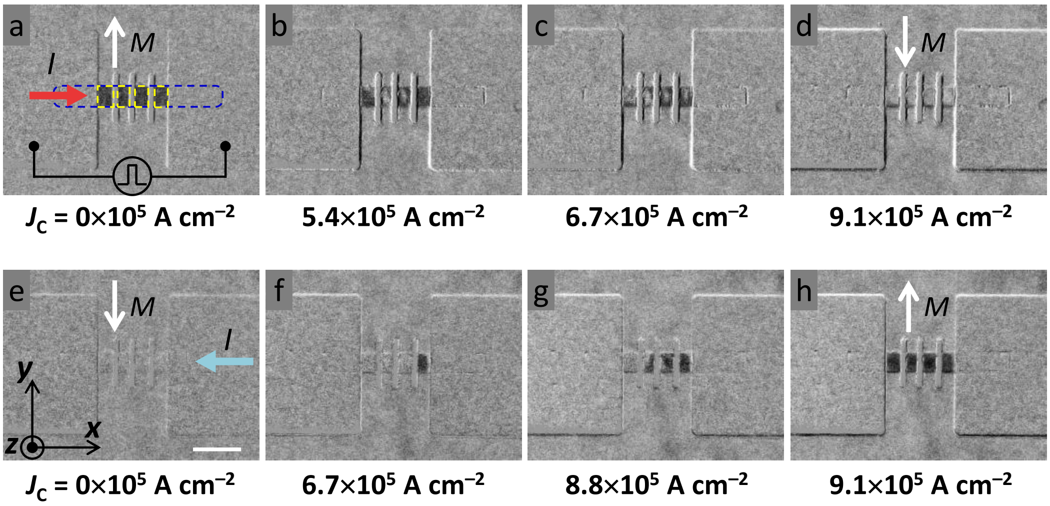

Supplementary Figure 16. MOKE images of SOT driven magnetization switching in Bi_2Se_3/NiO (1 nm)/Py at zero external magnetic field and room temperature. a-d, MOKE images for SOT driven magnetization switching using a pulsed current *I* along the +*x*-axis with increasing the current density J_C denoted underneath the corresponding image. **e-h**, MOKE images for SOT driven magnetization switching by *I* along the –*x*-axis. The dark (light) contrast shows the magnetization along the +*y* (–*y*)-axis, which is also indicated by the white arrows in (**a**), (**d**), (**e**) and (**h**). The white scale bar is 20 μm.

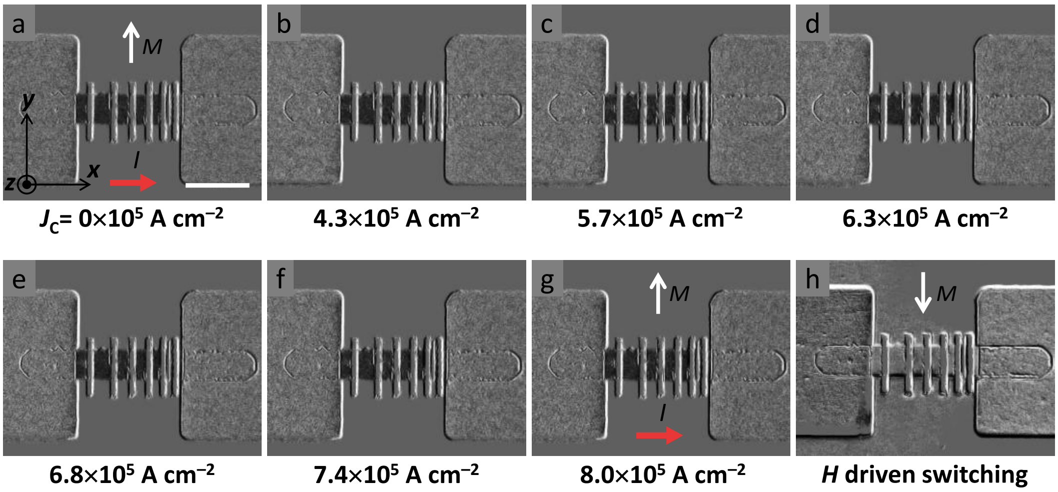

Supplementary Figure 17. MOKE images for Bi_2Se_3/NiO (2 nm)/Py at room temperature. a-g, MOKE images with increasing J_C, showing no current induced magnetization switching with the pulsed current I along +x-axis. The Py magnetization remains along +y-axis. The current channel is 10 μm wide and the pulsed current width is 500 μs. **h**, A reference MOKE image of H driven magnetization switching from +y to –y-axis, which is indicated by the change in contrast of the device in (**h**). The white scale bar is 20 μm.

Supplementary Note 1: HAADF-STEM image of Bi_2Se_3/NiFe heterostructures

The cross-section of the Bi_2Se_3/NiFe (Py) heterostructure is characterized with high-angle annular dark-field (HAADF) imaging in an aberration corrected scanning transmission electron microscope (STEM) as shown in Supplementary Fig. 1. We find that the Bi_2Se_3 has uniform and good layered structure, and one quintuple layer (QL) is around 1-nm thick. Moreover, the image shows a clean and smooth interface between the Bi_2Se_3 and Py layer.

Supplementary Note 2: Bi_2Se_3 thickness dependent sheet resistance, R_s

Supplementary Fig. 2 shows the sheet resistance, R_s, for different Bi_2Se_3 thicknesses. We find that R_s is small and remains almost constant at large thicknesses (15 and 20 QL). While R_s shows an abrupt increase as Bi_2Se_3 is thinner than 10 QL and it becomes maximum at 5 QL. This trend is similar as other reports[1,2].

Supplementary Note 3: Evaluation of conventional SOT efficiency (θ_{TI}) in Bi_2Se_3 by ST-FMR

As shown in Fig. 2c, we obtain the amplitudes of symmetric (V_S) and antisymmetric (V_A) components from the fits of the typical spin torque ferromagnetic resonance (ST-FMR) signal. Subsequently, the spin-orbit torque (SOT) efficiency θ_{TI} can be evaluated from only V_S by $V_S = -\frac{I_{RF}\gamma \cos\theta_H}{4}\frac{dR}{d\theta_H}\tau_{DL}\frac{1}{\Delta}F_S(H)$, $\sigma_S = J_S/E = \tau_{DL}M_s t/E$ and $\theta_{TI} = J_S/J_C = \sigma_S/\sigma$,[3,4] where I_{RF} is the RF current flowing through the device, γ is the gyromagnetic ratio, $dR/d\theta_H$ is the angular dependent magnetoresistance at $\theta_H = 35°$, Δ is the linewidth of ST-FMR signal, $F_S(H)$ is a symmetric Lorentzian, H is in-plane external magnetic field, τ_{DL} is the damping-like spin-orbit torque on unit $Co_{40}Fe_{40}B_{20}$ (CFB) moment at $\theta_H = 0°$, M_s is the saturation magnetization of CFB, t is the thickness of CFB, J_S is the measured spin current density with in-plane spin polarizations at the Bi_2Se_3/CFB interface, which is correlated with the measured symmetric component V_S as shown in Fig. 2c, J_C (A cm^{-2}) is the uniform charge current density in the Bi_2Se_3 layer, σ_S is the Bi_2Se_3 spin Hall conductivity, σ is the Bi_2Se_3 conductivity, and E is the microwave field across the device.

In the above *conventional* SOT efficiency evaluation method[3,4], the dimensionless θ_{TI} arising from the Bi_2Se_3 layer are obtained by using the uniform charge current density J_C (unit in A cm^{-2}) in the entire Bi_2Se_3 layer (including carriers from topological surface states,

two dimensional electron gas, and bulk states). It can represent the overall charge-to-spin conversion efficiency in the TI/FM device. On the other hand, it is of interest to reveal the *interface* SOT efficiency, λ_{TSS} (nm^{-1}), from only topological surface states (TSS) for which we need to use an interface charge current density $J_{C\text{-}TSS}$ (unit in A cm^{-1}) only in TSS (i.e. two dimensional carriers from TSS). λ_{TSS} from TSS is evaluated in Supplementary Note 6 and Supplementary Note 7.

Supplementary Note 4: Estimation of n_{TSS}, n_{2DEG}, $n_{2D\text{-}Bulk}$, E_F and k_F at different t_{BiSe}

On the basis of the t_{BiSe} dependent sheet carrier concentration, n_{2D}, in Fig. 1b, we estimate the contributions of three possible conduction channels to the electrical transport, such as surface states (including TSS and two dimensional electron gas, 2DEG) and bulk states (BS). As mentioned in the main text, the thickness of a TSS, t_{TSS}, and 2DEG, t_{2DEG}, in Bi_2Se_3 is reported to be ~1 nm and 4 nm, respectively. Therefore, no BS are expected if the Bi_2Se_3 thickness is less than ~8 QL (i.e. ≈ 8 nm). Consequently, n_{2D} can be writen as $n_{2D} = 2\times n_{TSS} + n_{2DEG}$ for $t_{BiSe} \le 8$ QL and $n_{2D} = 2\times n_{TSS} + n_{2DEG} + n_{2D\text{-}Bulk}$ for $t_{BiSe} > 8$ QL, where n_{TSS}, n_{2DEG} and $n_{2D\text{-}Bulk}$ are the sheet carrier concentration in a TSS layer, total 2DEG and BS, respectively, and the factor of 2 arises due to the bottom and top surfaces in Bi_2Se_3.

As shown in Supplementary Fig. 3, the TSS shows a linear band dispersion given by $E_F = \hbar V_F k_F + E_{DP}$, where E_F is the Fermi energy, V_F is Fermi velocity ~5×10^5 m s^{-1} in TSS,[5] $\hbar$ is reduced Plank constant, k_F is Fermi wave vector of TSS and E_{DP} is the energy of Dirac point (DP). Therefore, the density of states of TSS per surface area is linearly proportional to energy

$$D(E) = \frac{1}{2\pi}\frac{1}{(\hbar V_F)^2}(E - E_{DP}). \tag{1}$$

Then we can estimate n_{TSS} by

$$n_{TSS} = \int_{E_{DP}}^{\infty} D(E) f(E) d(E), \tag{2}$$

where $f(E) = 1/(1+e^{\frac{E-E_F}{K_B T}})$ is the Fermi-Dirac distribution function, K_B is Boltzmann constant and T is 300 K. Consequently, by substituting Supplementary Eq. 1 and f(E) into Supplementary Eq. 2, n_{TSS} can be rewritten as

$$n_{TSS} = \int_{E_{DP}}^{\infty} \frac{1}{2\pi}\left(\frac{K_B T}{\hbar V_F}\right)^2 \frac{\varepsilon}{1+e^{\varepsilon-\eta}} \mathrm{d}\varepsilon = \frac{1}{2\pi}\left(\frac{K_B T}{\hbar V_F}\right)^2 F_1(\eta), \tag{3}$$

where $\varepsilon = (E - E_{\mathrm{DP}})/K_B T$, $\eta = (E_{\mathrm{F}} - E_{\mathrm{DP}})/K_B T$ and $F_1(\eta)$ is the Fermi-Dirac integral of order 1. Under the condition of $E_{\mathrm{F}} - E_{\mathrm{DP}} \gg K_B T$, Supplementary Eq. 3 is simplified as

$$n_{\mathrm{TSS}} = \frac{1}{4\pi}\left(\frac{K_{\mathrm{B}} T}{\hbar V_{\mathrm{F}}}\right)^2 \eta^2 . \quad (4)$$

From Supplementary Eq. 4, we find that n_{TSS} is proportional to $(E_{\mathrm{F}} - E_{\mathrm{DP}})^2$. We can also estimate k_{F} by $k_{\mathrm{F}} = (E_{\mathrm{F}} - E_{\mathrm{DP}})/\hbar V_{\mathrm{F}}$.

The 2DEG is usually formed at the Bi_2Se_3 surface due to band bending. As shown in Supplementary Fig. 3, after band bending, E_{CBM} (E_{VBM}) evolves into a surface conduction band minimum, E_{SCBM} (surface valence band maximum, E_{SVBM}) at surface and E_{DP} shifts downwards to a higher binding energy. The quantum confinement along the film normal leads to the quantized subbands in 2DEG enclosed by TSS linear bands as shown in Supplementary Fig. 3. As the Bi_2Se_3 film is in the thick range ($t_{\mathrm{BiSe}} > 8$ QL), the TSS and the 2DEG on top and bottom surfaces are separated. The 2DEG subbands on each surface are respectively confined in a triangular quantum well caused by surface band bending[1,6]. As the Bi_2Se_3 film reduces below 8 QL, the 2DEG states from both surfaces merge, therefore, the square quantum confinement effects between the substrate and capping layer take over the triangular quantum well. It should be noted that, for clarity, the Rashba splitting is not illustrated in 2DEG subbands.

For $t_{\mathrm{BiSe}} \leq 8$ QL, a simple infinite square quantum well is assumed[1]. The energy minimum of each subband at $k_{||} = 0$ (E_{Cn}, $n = 1, 2\ldots$) gradually moves up with the energy E_{n} ($n = 1, 2\ldots$) away from E_{SCBM}, where $E_{\mathrm{n}} = \frac{\hbar^2\pi^2}{2m^* t_{\mathrm{BiSe}}{}^2} n^2$, where m^* is the effective mass of electron. m^* is ~0.14 m_0 for Bi_2Se_3,[7-9] where m_0 is the free-electron mass. The thinner the Bi_2Se_3 film, the more separation of 2DEG subbands, which indicates a decrease of the contribution of 2DEG subbands to the electrical transport as Bi_2Se_3 becomes thinner. For $t_{\mathrm{BiSe}} > 8$ QL, a simple triangular quantum well is used to describe the confined 2DEG subbands resulting from a band bending at each film surface[6]. The energy minimum of the allowed 2DEG subbands can be written as $E_{\mathrm{cn}} = E_{\mathrm{n}} + E_{\mathrm{SCBM}} = [\frac{3\pi}{2}(n - \frac{1}{4})]^{2/3} [\frac{\hbar^2 (\nabla V)^2}{2m^*}]^{1/3} + E_{\mathrm{SCBM}}$, where the integer n represents the quantum number of allowed 2DEG states and ∇V (meV Å^{-1}) is the potential gradient of triangular well near the Bi_2Se_3 surface, which can be estimated by the ratio of band bending energy (E_{CBM}-E_{SCBM}) and corresponding band bending depth along the film normal (~4 nm).

For each 2DEG subband, n_{2DEG} can be calculated by using the basic 2DEG equation $n_{2DEGn} = \frac{m^*}{\pi\hbar^2} K_B T \ln(1 + e^{(E_F - E_{Cn})/K_B T})$, where n = 1 and 2. We only consider the first and second subbands in our model since the third subband has a negligible contribution to n_{2DEG}. Note that for $t_{BiSe} \leq 8$ QL, the sheet carrier concentration n_{2DEG} in this model is from one 2DEG layer. However for $t_{BiSe} > 8$ QL, the sheet carrier concentration n_{2DEG} needs to consider two 2DEG layers.

The $n_{2D\text{-}Bulk}$ is estimated as follows. As it is known that the Bi_2Se_3 bulk can be regarded as a semiconductor with a band gap E_g ~0.3 eV as shown in Supplementary Fig. 3. The bulk carrier concentration is calculated by using the basic semiconductor equation[10] ($E_F - E_{CBM} > 3K_B$T, E_{CBM} is the bulk conductance band minimum)

$$n_{Bulk} = \frac{(2m^*)^{3/2}}{2\pi^2\hbar^3} \int_{E_c}^{\infty} \frac{(E - E_{CBM})^{1/2}}{1 + e^{[(E - E_F)/K_B T]}} \mathrm{d}E = N_C \frac{2}{\sqrt{\pi}} F_{1/2}(\xi), \quad (5)$$

where $N_C = 2(m^* K_B T / 2\pi\hbar^2)^{3/2}$. $F_{1/2}(\xi)$ is the Fermi-Dirac integral of order 1/2, where $\xi = (E_F - E_{CBM})/K_B T$. Then $n_{2D\text{-}Bulk}$ can be converted by $n_{2D\text{-}Bulk} = n_{Bulk} d$, where d is the bulk thickness excluding the TSS and 2DEG thicknesses. From Supplementary Eq. 5, we know that $n_{2D\text{-}Bulk}$ is related to $(E_F - E_{CBM})$.

In order to determine n_{TSS}, $n_{2D\text{-}bulk}$ and n_{2DEG}, we need to know the values of $E_F - E_{DP}$, $E_F - E_{CBM}$ and $E_F - E_{Cn}$, accordingly. On the basis of Supplementary Fig. 3, we simply rewrite $E_F - E_{Cn}$ as $E_F - E_{Cn} = (E_F - E_{SCBM}) - E_n = (E_F - E_{DP}) - (E_{SCBM} - E_{DP}) - E_n$. According to the reported ARPES data[5,11-13] and theoretical band structure calculation[14], we adopt $E_F - E_{CBM}$ ~100 meV and $E_{SCBM} - E_{DP}$ ~200 meV. Consequently, $E_F - E_{DP}$, which is determined by the magnitude of band bending induced by electron doping at surface, is the only variable to extract n_{2D} at different t_{BiSe}. $2 \times n_{TSS}$, n_{2DEG}, $n_{2D\text{-}Bulk}$, $E_F - E_{DP}$ and k_F of TSS as a function of t_{BiSe} are quantitatively determined and plotted in Fig. 4.

We have used $V_F = 5 \times 10^5$ m s^{-1}, $m^* = 0.14\ m_0$, $E_{SCBM} - E_{DP} = 200$ meV and $E_F - E_{CBM} =$ 100 meV for the estimation in Fig. 4 in the main text. Additionally, we have estimated n_{2D} by varying the parameters with the range from literatures, including V_F, m^*, $E_{SCBM} - E_{DP}$ and $E_F - E_{CBM}$, respectively. First, we vary the value of V_F from 4.6×10^5 to 5.4×10^5 m s^{-1},[5,15-17] and fix $m^* = 0.14\ m_0$, $E_{SCBM} - E_{DP} = 200$ meV and $E_F - E_{CBM} = 100$ meV. The estimated results are shown in Supplementary Fig. 4. We find that as the V_F increases, the n_{TSS} only slightly decreases. Moreover, the n_{2D} keeps the similar trend as that in the Fig. 4a (also see

Supplementary Fig. 4b). Overall, the value of V_F does not affect the estimation results significantly.

Similarly, we varied the value of m^* in the range of $0.11m_0$ to $0.17m_0$,[6-9,12,13,18] and fixed $V_F = 5\times10^5$ m s^{-1}, $E_{SCBM} - E_{DP}$ = 200 meV and $E_F - E_{CBM}$ = 100 meV as used in the main text. As shown in Supplementary Fig. 5, we find that as m^* increases, the n_{2DEG} and $n_{2D\text{-}Bulk}$ only slightly increase. Moreover, the results in Supplementary Fig. 5 are similar to the values shown in the main text.

In addition, we also varied the value of $E_{SCBM} - E_{DP}$ from 150 to 250 meV,[17,19-21] and the value of $E_F - E_{CBM}$ from 50 to 150 meV.[5,11,12,17,19] The results are shown in Supplementary Fig. 6 and 7, respectively. We find that different parameters can only weakly affect the values of n_{TSS}, n_{2DEG} and $n_{2D\text{-}Bulk}$, and the main features of the results in the main text still hold good.

Supplementary Note 5: Current shunting (I_{TSS}/I_{total}) at different t_{BiSe}

The current flowing in each channel (TSS, 2DEG, and BS) can be written as $I_{TSS} = n_{TSS}\mu_{TSS}eEW$, $I_{2DEG} = n_{2DEG}\mu_{2DEG}eEW$ and $I_{Bulk} = n_{2D\text{-}Bulk}\mu_{Bulk}eEW$ respectively, where W and E are the channel width and electric field (same for TSS, 2DEG and BS channels) in each device, and μ_{TSS}, μ_{2DEG} and μ_{Bulk} are the carrier mobility in TSS, 2DEG and BS, respectively. The linear Hall curves we measured at different t_{BiSe} indicate the carriers in Bi_2Se_3 have a similar mobility[1]. Thus, we obtain I_{TSS}/I_{total} at different t_{BiSe} by assuming $\mu_{TSS} = \mu_{2DEG} = \mu_{Bulk}$ (Fig. 4c in the main text), where I_{total} is the total current flowing in a Bi_2Se_3 film.

Supplementary Note 6: Evaluation of the interface SOT efficiency, λ_{TSS}, from TSS

As discussed in Supplementary Note 3, the θ_{TI} versus t_{BiSe} from ST-FMR measurements is obtained by using a uniform charge current density J_C (A cm^{-2}) in the entire Bi_2Se_3 layer as $\theta_{TI} = \frac{J_S}{J_C} = \frac{J_S}{n_{2D}\mu eE/t_{BiSe}}$. The *interface* SOT efficiency from only TSS, λ_{TSS} (nm^{-1}), can be obtained by the *interface* charge current density $J_{C\text{-}TSS}$ (A cm^{-1}) in TSS as $\lambda_{TSS} = \frac{J_S}{J_{C\text{-}TSS}} = \frac{J_S}{n_{TSS}\mu eE}$. Therefore, we can evaluate λ_{TSS} by $\lambda_{TSS} = \frac{n_{2D}/t_{BiSe}}{n_{TSS}}\theta_{TI}$. The estimated λ_{TSS} is plotted in Fig. 4d.

Supplementary Note 7: Estimation of λ_{2DEG} in 2DEG and λ_{intTSS}

According to above analysis, for $t_{BiSe} \leq 8$ QL, there are only TSS and 2DEG contribution to the SOT efficiency. As mentioned in the main text, the spin current density at the interface arising from only TSS ($J_{S\text{-}TSS}$) is not necessarily equal to the spin current density (J_S) from ST-FMR measurements due to the partial cancellation by the opposite spin polarizations from Rashba splitting in 2DEG. Based on this scenario, we rewrite the λ_{TSS} in Fig. 4d as $\lambda_{TSS} = \frac{J_{S\text{-}TSS} - J_{S\text{-}2DEG}}{J_{C\text{-}TSS}} = \frac{J_{S\text{-}TSS}}{J_{C\text{-}TSS}} - \frac{J_{S\text{-}2DEG}}{J_{C\text{-}TSS}}$, where $J_{S\text{-}TSS}/J_{C\text{-}TSS}$ is the intrinsic interface SOT efficency from TSS ($\lambda_{intriTSS}$) which is inversely propotional to V_F and almost remain constant at different t_{BiSe}[22], and $J_{S\text{-}2DEG}$ is the spin current density from Rashba splitting in 2DEG. Then we get $\lambda_{TSS} = \lambda_{intriTSS} - \frac{\lambda_{2DEG} J_{C\text{-}2DEG}}{J_{C\text{-}TSS}}$, where λ_{2DEG} is the interface SOT efficiency from Rashba splitting in 2DEG, $J_{C\text{-}2DEG} = n_{2DEG}\mu eE$ and $J_{C\text{-}TSS} = n_{TSS}\mu eE$. We assume that the difference of surface band bending between 7 and 8-QL Bi_2Se_3 films is small, which results in an almost constant λ_{2DEG}. By using the difference of λ_{TSS} between 7 and 8 QL film as shown in Fig. 4d, the λ_{2DEG} is determined and it shows negative value and is ~ –0.4 nm^{-1}. Moreover, the values for $\lambda_{intriTSS}$ are also estimated for $t_{BiSe} \leq 10$ QL with negligible BS. Interestingly, we find $\lambda_{intriTSS}$ shows a constant value of ~0.8 nm^{-1} for 7, 8 and 10 QL Bi_2Se_3 films. This amended interface SOT efficiency is in the simliar range of the value of λ_{TSS} (~0.82 nm^{-1}) at $t_{BiSe} = 5$ QL as shown in Fig. 4d. This further substantiates our claim of TSS dominated SOT in thinner films and the high SOT efficiency from TSS.

We have used $V_F = 5\times10^5$ m s^{-1}, $m^* = 0.14\ m_0$, $E_{SCBM} - E_{DP} = 200$ meV and $E_F - E_{CBM} = 100$ meV for the estimation in Fig. 4 in the main text. Additionally, similar to Supplementary Note 4, we have also estimated the interface SOT efficiency, λ_{TSS} and amended λ_{TSS} by varying the parameters V_F, m^*, $E_{SCBM} - E_{DP}$ and $E_F - E_{CBM}$ with the range of values from literatures. First, we varied the value of V_F from 4.6×10^5 to 5.4×10^5 m s^{-1},[5,15-17] and fixed $m^* = 0.14\ m_0$, $E_{SCBM} - E_{DP} = 200$ meV and $E_F - E_{CBM} = 100$ meV. The estimated results are shown in Supplementary Fig. 8. We find that as the V_F varies, the interface SOT efficiencies do not change much and show similar values with respect to that in the Fig. 4d (also see Supplementary Fig. 8b). Overall, the V_F does not affect the estimation results significantly.

Similarly, we varied the value of m^* in the range of $0.11m_0$ to $0.17m_0$,[6-9,12,13,18] and fixed $V_F = 5\times10^5$ m s^{-1}, $E_{SCBM} - E_{DP} = 200$ meV and $E_F - E_{CBM} = 100$ meV as used in the main text.

As shown in Supplementary Fig. 9, we find that as m^* increases, the interface SOT efficiency only slightly increases and the results in Supplementary Fig. 9 are similar to the values shown in the main text.

In addition, we also varied the value of $E_{SCBM} - E_{DP}$ from 150 to 250 meV,[17,19-21] and the value of $E_F - E_{CBM}$ from 50 to 150 meV.[5,11,12,17,19] The results are shown in Supplementary Fig. 10 and 11, respectively. We find different parameters can only weakly affect the values of the interface SOT efficiency and the main features of the results in the main text still hold good.

Supplementary Note 8: Reproducible SOT driven magnetization switching in Bi_2Se_3/NiFe device

The SOT driven magnetization switching by currents is reproducible in other Bi_2Se_3 (8 QL)/Py (6 nm) devices. One example is shown in Supplementary Fig. 12. We use the same measurement condition used in Fig. 5 and the current channel is 12 μm wide. We find that the switching current density is ~6×10^5 A cm^{-2}, which is similar as that in the device in Fig. 5.

Supplementary Note 9: SOT efficiency from current induced magnetization switching

For the conventional antidamping spin torque driven magnetization switching, the critical switching current density J_{C0} for the switching scheme of our Bi_2Se_3/Py device can be described by[23,24]

$$J_{C0} = \frac{2e}{\hbar}\mu_0 M_s t\alpha(H_c + M_{eff}/2)/\theta_{TI}, \qquad (6)$$

where J_{C0} is the critical switching current density without thermal fluctuation, M_s, t, α, H_c and M_{eff} are the saturated magnetization, thickness, damping constant, coercive field and effective magnetization of Py layer, respectively, and θ_{TI} is the SOT efficiency. The Supplementary Eq. 6 is based on the macrospin model in the absence of thermal fluctuation. In our device, the magnetization switching process can be described by the localized nucleation of reverse domains with an activation volume V_N first, followed by domain wall propagation. We anticipate that the magnetization exhibits coherent reversal inside the activation volume V_N. Therefore, the Supplementary Eq. 6 can apply in our device by introduction of V_N instead of the whole volume of Py layer. In our measurements, the switching current density J_C for the magnetization switching is ~6.2×10^5 A cm^{-2} at room temperature. Then the J_{C0} can be

obtained by $\frac{J_{C}}{J_{C0}} = 1 - \frac{K_{B}T}{K_{Py}V_{N}} \ln \frac{t_{P}}{t_{0}}$ with thermal fluctuation consideration[25-27], where t_P is the current pulse width ~500 μs, t_0 is the attempt time ~1 ns, the anisotropy energy density K_{Py} is estimated by $H_cM_s/2$ with measured H_c ~6.9 Oe and $M_s = 6.84\pm0.03\times10^5$ A m^{-1}. The domain wall width δ_m of Py layer is assumed to be ~220 nm,[28,29] and t is 6 nm, then we can estimate $V_N \approx \delta_m^2 t$. Consequently, we find J_{C0} ~$5.26\times J_C$. The M_{eff} and α are ~0.57 T and ~0.01543, respectively, which are obtained from ST-FMR and vibrating sample magnetometer (VSM) measurements. From Supplementary Eq. 6, we determine the SOT efficiency θ_{TI} for Bi_2Se_3/Py to be ~1.71. This value is consistent with the value obtained from ST-FMR measurements (θ_{TI} ~1).

The charge to spin conversion efficiency is typically less than one from the definition of $\theta_{TI} = J_S/J_C$. However, recently, there have been several reports to evaluate θ_{TI} in the topological insulators (TIs) and they have observed θ_{TI} greater than one. Specifically, the θ_{TI} values are reported to be ~2–3.5 (in Bi_2Se_3/Py by ST-FMR measurements at room temperature)[3], ~140–425 (in Cr doped BiSbTe/$(Bi_{0.5}Sb_{0.5})_2Te_3$ bilayer by magnetization switching at 1.9 K)[30] and ~20 (in $(Bi_{0.5}Sb_{0.5})_2Te_3$ by spin tunneling spectroscopy method below 200 K)[31]. Therefore, we speculate that the spin-momentum locking at the TSS in TIs plays an important role. Additionally, the recent reported multi-cycle spin transfer scenario[32] could be also responsible for a large θ_{TI}, which is explained as follows. As a charge current flows within Bi_2Se_3 TI film, spin accumulation is generated and the spins flow vertically toward the ferromagnetic Py layer. These spins are absorbed and exert SOTs on the Py magnetization. The θ_{TI} is evaluated by the measured torque in the ST-FMR measurements. Microscopically, in this process, electrons become spin polarized at the TSS due to spin-momentum locking and transfer the spin angular momentum to the Py magnetization at the interface of Bi_2Se_3/Py. However, these electrons can again diffuse back into the Bi_2Se_3 layer due to no net charge current flowing vertically into the Py layer. This above process repeats multiple times. Therefore, each electron can transfer the spin angular momentum multiple times, as it moves opposite to the charge current direction, and thus exert large torques on the Py layer. As reported recently[15,33-36], the electrons can be spin polarized in Bi_2Se_3 TSS with very large spin polarization values ranging from ~0.2 to 0.75 due to the spin momentum locking. Therefore, we think the large spin polarization in TSS and the multi-cycle spin transfer scenario are the possible reasons for the θ_{TI} greater than one observed in TIs.

Supplementary Note 10: Current polarity dependent magnetization switching

Supplementary Fig. 13 shows that the current induced magnetization switching depends on the charge current polarity which is a characteristic of SOT driven magnetization switching. For these measurements, we use the same device as in Fig. 5. In Supplementary Fig. 13, the Py magnetization direction in each case is indicated by a white arrow. For the set of measurements in Supplementary Fig. 13a-c, we first saturate the Py magnetization along the +*y*-axis by applying an in-plane external magnetic field (*H*). Then we remove *H* and capture the corresponding MOKE image as the initialized state as shown in Supplementary Fig. 13a. The dark contrast represents the magnetization along the +*y*-axis, which is indicated by the white arrow. Subsequently, we apply a pulsed current *I* along the –*x*-axis with current density $J_C = 6.2\times10^5$ A cm^{-2} without an external magnetic field (see Supplementary Fig. 13b). We observe that there is no magnetization switching as the magnetization remains along its initial +*y*-axis indicated by the no contrast change. However, as we change the current polarity by applying *I* along the +*x*-axis with the same value of J_C, we observe that the Py magnetization switches from the +*y* to –*y*-axis indicated by the light contrast (see Supplementary Fig. 13c).

Similarly, for the other set of measurements in Supplementary Fig. 13d-f, we first initialize the Py magnetization along the –*y*-axis. Then we remove *H* and capture the corresponding MOKE image (see Supplementary Fig. 13d). Subsequently, we apply the current pulses of different polarities with $J_C = 6.2\times10^5$ A cm^{-2}. We find that only the current pulse along the –*x*-axis can realize magnetization switching from the –*y* to +*y*-axis indicated by the dark contrast (see Supplementary Fig. 13f). The dependence of magnetization switching direction on the polarity of current suggests that the switching in our case is primarily driven by the SOT and not by any thermal effects.

Supplementary Note 11: SOT induced magnetization switching with Cu and NiO insertions

In order to further verify the SOT induced magnetization switching in Bi_2Se_3/Py, we have performed the MOKE imaging measurements with the same condition used in Fig. 5 on the control devices of Bi_2Se_3 (8 QL)/insertion layer/Py (6 nm)/MgO (1 nm)/SiO_2 (4 nm), where the insertion layer is Cu (1 and 2 nm) or insulating layer NiO (1 nm).

It is well known that Cu has a very low spin-orbit coupling strength and a long spin diffusion length. Hence, Cu does not possibly affect the spin generation in the Bi_2Se_3 layer and can be a good spin conductor. We follow the recent report[22] and use Cu insertion to

separate the Bi_2Se_3 and Py layer. The Cu is sputtered on top of Bi_2Se_3 with a low power of 30 W. As shown in Supplementary Fig. 14, the SOT driven magnetization switching is observed in Bi_2Se_3 (8 QL)/Cu (1 nm)/Py (6 nm) devices. We find that the switching current density J_C is ~6.7×10^5 A cm^{-2}, which is similar as that in the device without Cu insertion (~6.2×10^5 A cm^{-2}) in Fig. 5 and Supplementary Fig. 12. Similarly, Supplementary Fig. 15 reveals that the SOT driven magnetization switching by currents can also be observed in Bi_2Se_3 (8 QL)/Cu (2 nm)/Py (6 nm) devices. The switching current density slightly increases and is ~7.7×10^5 A cm^{-2}, which might be due to a bit of spin scattering in the Cu insertion layer. The devices with 1–2 nm Cu insertion exhibit almost similar device resistance as that with no Cu insertion from an independent four probe measurements. This indicates that the very thin Cu insertion has a much higher resistivity than the Py layer which is expected and is a usual case for very thin films. Therefore, for simplicity, we do not consider the current shunting in the Cu insertion and take the upper bound of the J_C denoted in the Supplementary Fig. 14 and 15.

In addition, we also perform the MOKE imaging measurements on the devices with insulating NiO insertion between the Bi_2Se_3 and Py layer. As shown in Supplementary Fig. 16, the SOT driven magnetization switching is observed in Bi_2Se_3 (8 QL)/NiO (1 nm)/Py (6 nm) devices. We find that the switching current density increases compared to that in Fig. 5. The magnetization starts to switch at J_C ~5.4–6.7 $\times10^5$ A cm^{-2} and fully switches at J_C ~9.1×10^5 A cm^{-2}. This is expected since the spins are blocked by an insulator and the transmission will potentially decrease as the insulator thickness increases. As shown in Supplementary Fig. 17, we cannot observe SOT induced magnetization switching with J_C up to ~8 $\times10^5$ A cm^{-2} as the NiO insertion layer becomes 2 nm.

By inserting Cu and insulating NiO layers, we can (at least partially) prevent the direct interface between Bi_2Se_3 and Py. The SOT induced magnetization switching by Bi_2Se_3 is reproducible and robust in different devices. Therefore, we further confirm the highly efficient SOT induced magnetization switching in our Bi_2Se_3/Py heterostructures.

Supplementary Note 12: Influence of Oersted field on current induced magnetization switching

We perform the MOKE imaging measurements on a control device of Bi_2Se_3 (8 QL)/NiO (2 nm)/Py (6 nm)/MgO (1 nm)/SiO_2 (4 nm) with the same condition used in Fig. 5. Due to the 2-nm NiO insulating insertion layer, the spin currents propagating from Bi_2Se_3 into Py is attenuated significantly, which is confirmed by the ST-FMR measurements. Therefore, the

current induced Oersted field (H_{Oe}) would be the main driving force on the Py layer. At the beginning of this set of measurements, we first saturate the Py magnetization along the +*y*-axis by applying an in-plane external magnetic field (*H*). Then we remove *H* and apply *I* along the +*x*-axis to the device. When the current density in Bi_2Se_3 (J_C) is zero, we capture the MOKE image as shown in Supplementary Fig. 17a. The dark contrast represents the magnetization along the +*y*-axis, which is indicated by the white arrow. Then we gradually increase J_C at room temperature without *H*. As shown in Supplementary Fig. 17b-g, there is no current induced magnetization switching as the magnetization is still along the +*y*-axis indicated by the no contrast change. In these measurements, the applied J_C is up to 8×10^5 A cm^{-2} (Supplementary Fig. 17g) which is almost 3 times larger than the ones at which the magnetization switching is triggered in the devices in Fig. 5 and Supplementary Fig. 12. Supplementary Fig. 17h shows a reference MOKE image of magnetization switching from +*y* to –*y*-axis driven by an applied external magnetic field *H*, which is indicated by the change in contrast to light colour. Moreover, the H_{Oe} from Bi_2Se_3 layer is estimated to be ~0.12–0.3 Oe by the equation[37] $H_{Oe} = J_C t_{BiSe}/2$. We find that even this calculated value of H_{Oe} is much smaller than the required switching field of Py layer. Therefore, the H_{Oe} is not the mechanism for the current induced magnetization switching we observed in the Bi_2Se_3/Py devices.

Supplementary References

1 Bansal, N., Kim, Y. S., Brahlek, M., Edrey, E. & Oh, S. Thickness-Independent Transport Channels in Topological Insulator Bi_2Se_3 Thin Films. *Phys. Rev. Lett.* **109**, 116804 (2012).
2 Taskin, A., Sasaki, S., Segawa, K. & Ando, Y. Manifestation of topological protection in transport properties of epitaxial Bi_2Se_3 thin films. *Phys. Rev. Lett.* **109**, 066803 (2012).
3 Mellnik, A. *et al.* Spin-transfer torque generated by a topological insulator. *Nature* **511**, 449-451 (2014).
4 Wang, Y. *et al.* Topological Surface States Originated Spin-Orbit Torques in Bi_2Se_3. *Phys. Rev. Lett.* **114**, 257202 (2015).
5 Xia, Y. *et al.* Observation of a large-gap topological-insulator class with a single Dirac cone on the surface. *Nat. Phys.* **5**, 398-402 (2009).
6 Benia, H. M., Lin, C., Kern, K. & Ast, C. R. Reactive Chemical Doping of the Bi_2Se_3 Topological Insulator. *Phys. Rev. Lett.* **107**, 177602 (2011).
7 Wang, Z. *et al.* Tuning carrier type and density in Bi_2Se_3 by Ca-doping. *Appl. Phys. Lett.* **97**, 042112 (2010).
8 Lawson, B. J., Hor, Y. S. & Li, L. Quantum Oscillations in the Topological Superconductor Candidate $Cu_{0.25}Bi_2Se_3$. *Phys. Rev. Lett.* **109**, 226406 (2012).
9 Cao, H. *et al.* Quantized Hall Effect and Shubnikov-de Haas Oscillations in Highly Doped Bi_2Se_3: Evidence for Layered Transport of Bulk Carriers. *Phys. Rev. Lett.* **108**, 216803 (2012).

10 Sze, S. M. & Ng, K. K. *Physics of Semiconductor Devices, 3rd Edition*. (John Wiley & Sons, 2006).
11 Bahramy, M. S. *et al.* Emergent quantum confinement at topological insulator surfaces. *Nat. Commun.* **3**, 1159 (2012).
12 Bianchi, M. *et al.* Coexistence of the topological state and a two-dimensional electron gas on the surface of Bi_2Se_3. *Nat. Commun.* **1**, 128 (2010).
13 Analytis, J. G. *et al.* Bulk Fermi surface coexistence with Dirac surface state in Bi_2Se_3: A comparison of photoemission and Shubnikov-de Haas measurements. *Phys. Rev. B* **81**, 205407 (2010).
14 Liu, Y. *et al.* Tuning Dirac states by strain in the topological insulator Bi_2Se_3. *Nat. Phys.* **10**, 294-299 (2014).
15 Yazyev, O. V., Moore, J. E. & Louie, S. G. Spin polarization and transport of surface states in the topological insulators Bi_2Se_3 and Bi_2Te_3 from first principles. *Phys. Rev. Lett.* **105**, 266806 (2010).
16 Zhang, H. *et al.* Topological insulators in Bi_2Se_3, Bi_2Te_3 and Sb_2Te_3 with a single Dirac cone on the surface. *Nat. Phys.* **5**, 438 (2009).
17 Kuroda, K. *et al.* Hexagonally Deformed Fermi Surface of the 3D Topological Insulator Bi_2Se_3. *Phys. Rev. Lett.* **105**, 076802 (2010).
18 Butch, N. P. *et al.* Strong surface scattering in ultrahigh-mobility Bi_2Se_3 topological insulator crystals. *Phys. Rev. B* **81**, 241301 (2010).
19 Jozwiak, C. *et al.* Photoelectron spin-flipping and texture manipulation in a topological insulator. *Nat. Phys.* **9**, 293-298 (2013).
20 Chang, J., Register, L. F. & Banerjee, S. K. Topological insulator Bi_2Se_3 thin films as an alternative channel material in metal-oxide-semiconductor field-effect transistors. *J. Appl. Phys.* **112**, 124511 (2012).
21 Romanowich, M. *et al.* Interplay of topological surface and bulk electronic states in Bi_2Se_3. *Phys. Rev. B* **87**, 085310 (2013).
22 Kondou, K. *et al.* Fermi-level-dependent charge-to-spin current conversion by Dirac surface states of topological insulators. *Nat. Phys.* **12**, 1027-1031 (2016).
23 Liu, L. *et al.* Spin-Torque Switching with the Giant Spin Hall Effect of Tantalum. *Science* **336**, 555 (2012).
24 Fukami, S., Anekawa, T., Zhang, C. & Ohno, H. A spin–orbit torque switching scheme with collinear magnetic easy axis and current configuration. *Nat. Nanotech.* **11**, 621-625 (2016).
25 Zhitao, D. *et al.* Spin-transfer torque switching in magnetic tunnel junctions and spin-transfer torque random access memory. *J. Phys.: Condens. Matter* **19**, 165209 (2007).
26 Hosomi, M. *et al.* in *Electron Devices Meeting, 2005. IEDM Technical Digest. IEEE International.* 459-462 (IEEE).
27 Chun, K. C. *et al.* A scaling roadmap and performance evaluation of in-plane and perpendicular MTJ based STT-MRAMs for high-density cache memory. *IEEE J. Solid-State Circuits* **48**, 598-610 (2013).
28 Wong, B. Y. & Laughlin, D. E. Direct observation of domain walls in Py films using high-resolution Lorentz microscopy. *J. Appl. Phys.* **79**, 6455-6457 (1996).
29 Weinberger, P. Domain Wall Formation in Ni_xFe_{1-x}. *Phys. Rev. Lett.* **98**, 027205 (2007).
30 Fan, Y. *et al.* Magnetization switching through giant spin–orbit torque in a magnetically doped topological insulator heterostructure. *Nat. Mater.* **13**, 699-704 (2014).
31 Liu, L. *et al.* Spin-polarized tunneling study of spin-momentum locking in topological insulators. *Phys. Rev. B* **91**, 235437 (2015).

32 Hellman, F. *et al.* Interface-induced phenomena in magnetism. *Rev. Mod. Phys.* **89**, 025006 (2017).
33 Li, C. *et al.* Electrical detection of charge-current-induced spin polarization due to spin-momentum locking in Bi_2Se_3. *Nat. Nanotech.* **9**, 218-224 (2014).
34 Dankert, A., Geurs, J., Kamalakar, M. V., Charpentier, S. & Dash, S. P. Room temperature electrical detection of spin polarized currents in topological insulators. *Nano Lett.* **15**, 7976-7981 (2015).
35 Neupane, M. *et al.* Observation of quantum-tunnelling-modulated spin texture in ultrathin topological insulator Bi_2Se_3 films. *Nat. Commun.* **5**, 3841 (2014).
36 Pan, Z. H. *et al.* Electronic Structure of the Topological Insulator Bi_2Se_3 Using Angle-Resolved Photoemission Spectroscopy: Evidence for a Nearly Full Surface Spin Polarization. *Phys. Rev. Lett.* **106**, 257004 (2011).
37 Liu, L., Moriyama, T., Ralph, D. C. & Buhrman, R. A. Spin-Torque Ferromagnetic Resonance Induced by the Spin Hall Effect. *Phys. Rev. Lett.* **106**, 036601 (2011).
